# Prognostic impact of miR-17-5p and miR-20a-5p in NSCLC diverge in subgroups according to lymph node status

**DOI:** 10.3389/fonc.2025.1606933

**Published:** 2025-09-25

**Authors:** Dagny Førde, Thomas Kilvær, Mona Irene Pedersen, Ana Paola Lombardi, Irene D’arsiè, Erna-Elise Paulsen, Lill-Tove Rasmussen Busund, Mehrdad Rakaee, Tom Dønnem, Sigve Andersen

**Affiliations:** ^1^ Translational Cancer Research Group, Institute of Clinical Medicine, UiT The Arctic University of Norway, Tromsø, Norway; ^2^ Department of Oncology, University Hospital of North Norway, Tromsø, Norway; ^3^ Translational Cancer Research Group, Institute of Medical Biology, UiT The Arctic University of Norway, Tromsø, Norway; ^4^ Cellular, Computational and Integrative Biology, University of Trento, Trento, Italy; ^5^ Department of Clinical Pathology, University Hospital of North Norway, Tromsø, Norway; ^6^ Department of Cancer Genetics, Oslo University Hospital, Oslo, Norway

**Keywords:** NSCLC, miR-17, miR-20, prognosis, cell lines, digital pathology

## Abstract

**Introduction:**

MicroRNAs (miRs) are short non-coding, functional RNA molecules that regulate gene expression. Different miRs are frequently dysregulated and implicated in the development and outcome of non-small cell lung cancer (NSCLC). We investigated the prognostic and functional aspects of miR-17-5p and miR-20a-5p by.

**Methods:**

*in situ* hybridization in a large, well-characterized cohort of resected NSCLC patients and through overexpression in two NSCLC cell lines.

**Results:**

In the overall cohort, we observed no prognostic impact of miR-17-5p and miR-20a-5p in univariate analyses, while high expression of miR-20a-5p was associated with a positive outcome in multivariate analyses (HR 0.732, 95% CI 0.544–0.986, p = 0.040). In subgroup analyses, high expression of miR-20a-5p was associated with a positive prognosis in patients with lung squamous cell carcinoma and lymph node metastases (N+). Interestingly, miR-17-5p was associated with a poor prognosis in patients without lymph node metastases (N0), while no prognostic impact was observed in N+ patients. In cell line studies, overexpression of miR-17-5p did not influence proliferation but led to increased invasion in both investigated cell lines. Overexpression of miR-20a-5p led to decreased proliferation in one of two investigated cell lines and, like miR-17-5p, increased invasion.

**Discussion:**

Overall, our results suggest that the prognostic role of miR-17-5p and miR-20a-5p in early-stage NSCLC is context-dependent. Consequently, further studies are needed to elucidate the role of these miRs during NSCLC carcinogenesis. Clinical implementation should not be initiated until their role in different disease settings is sufficiently understood.

## Introduction

Lung cancer is the leading cause of cancer-related morbidity and mortality worldwide and is responsible for as many life-years lost as colorectal, breast, and prostate cancers combined (1). Non-small cell lung cancer (NSCLC) represents approximately 85% of all lung cancer cases (2). As with most solid tumors, prognostication and treatment stratification for NSCLC patients are primarily guided by the TNM system, which evaluates tumor size and local invasion (T), lymph node involvement (N), and distant metastasis (M) (3, 4). However, as clinical outcomes vary significantly within each stage, it is evident that TNM staging alone provides incomplete prognostic information. In recent years, molecular and histopathological features, including mutational status and immune-related biomarkers, have gained increasing importance for personalized treatment strategies (5). Additional insights into functional aspects of tumor cells and their microenvironment may further refine prognostication and guide treatment decision-making, ultimately improving patient outcomes.

MicroRNAs (miRs) are short, non-coding, functional RNA molecules, with a length of 19–25 nucleotides, known to regulate gene expression post-transcriptionally by repressing translation or via targeted messenger RNA (mRNA) degradation (6). Aberrant miR expression is a hallmark of many cancers, where they can promote or suppress oncogenesis (7). These alterations can result from genomic amplification or deletion, transcriptional dysregulation, epigenetic modifications, or defects in the miR biogenesis machinery (8), leading to the dysregulation of critical oncogenes and tumor suppressors. Genome-wide profiling studies have demonstrated that miR signatures can distinguish between cancer types (9). Moreover, due to their remarkable stability in tissues and body fluids (10–12), miRs are increasingly being explored as non-invasive cancer biomarkers and have even reached phase I clinical trials (12, 13). A recent meta-analysis reported on the diagnostic potential of circulating miR-17-5p for the detection of NSCLC within the Chinese population (14).

Numerous tumor-suppressing and tumor-promoting miRs have been implicated in lung cancer (15). Of particular interest, results from studies in cell lines and mice suggest that miR-targeted therapies may be feasible (16). The members of the miR-17–92 cluster, collectively referred to as “onco-miR-1” due to their frequent overexpression in multiple cancer types, including NSCLC, are among the most studied miRs (17). This cluster regulates several oncogenic pathways such as PI3K/AKT, p53 signaling, EGFR, and cell cycle signaling (18). Notably, miR-17-5p and miR-20a-5p, key members of this cluster, play central roles in cell cycle regulation by targeting the E2F transcription factor, affecting cell cycle progression and MYC signaling (19–21). In NSCLC, Matsubara et al. showed that cells overexpressing miR-17–92 underwent apoptosis when treated with antisense oligonucleotides against miR-17-5p and miR-20a-5p (22). However, while earlier studies broadly classified the miR-17–92 cluster as oncogenic (17, 18), recent findings suggest a context-dependent role. MiR-17-5p has been implicated in reducing tumorigenicity and modulating immune evasion via the regulation of RUNX3 and long non-coding RNAS such as FGD5-AS1 (16, 23, 24), indicating that its function may vary depending on tumor subtype, stage, or cellular context. In contrast, miR-20a-5p remains less extensively studied, although it is also involved in key oncogenic processes. Gont et al. suggested that miR-20a-5p acts as an oncogene by downregulating PTEN and increasing PD-L1 expression (25), while others report tumor-suppressive functions, including the inhibition of proliferation and angiogenesis (26).

Despite these insights, the prognostic and functional relevance of miR-17-5p and miR-20a-5p in NSCLC remains unclear, largely due to conflicting findings in cell lines and a lack of high-quality, large, tissue-based studies integrating expression data with clinical outcomes. Prior prognostic studies have primarily used qRT-PCR to analyze these miRs in circulating cells and not in NSCLC tissue (27), which may not accurately capture intratumoral biology. To address this gap, we aim to explore the functional and clinical impact of miR-17-5p and miR-20a-5p expression in NSCLC through 1) characterization of their value as prognostic biomarkers in a large, described cohort of resected NSCLC patients with an extensive follow-up and 2) cell line experiments to assess their biological function.

## Materials and methods

### Patients

The study population comprised 633 consecutive stage I to IIIB NSCLC patients who underwent curative-intent radical resection at the University Hospital of North Norway or the Nordland Central Hospital between 1990 and 2010. Of these, 80 patients were excluded due to neoadjuvant radio-chemotherapy, other malignancy within 5 years before NSCLC diagnosis, inadequate tissue in paraffin-embedded formalin-fixed blocks, or poor tissue quality. Consequently, 553 patients were available for analysis. All patients were restaged according to the 8th edition of the Union for International Cancer Control (UICC) TNM classification system (4). The median follow-up of survivors was 86 months (range 34–267 months). Follow-up data were last updated October 1, 2013. Detailed information regarding the study population has been previously published (28).

### Tissue samples and tissue microarray construction

Formalin-fixed and paraffin-embedded (FFPE) tumor and control specimens were obtained from the hospital archives. An experienced pathologist reviewed the hematoxylin and eosin (H&E)-stained slides of the face of all FFPE blocks and marked representative tumor and stromal areas. A tissue-arraying instrument (Beecher Instruments, Silver Spring, MD, USA) was used to make tissue microarrays (TMAs). Four replicate tissue cores, each 0.6 mm, were transferred from each donor block to a recipient block. The procedure in detail has been previously reported (29). Sections were cut at 4 µm using Microm microtome HM355S for H&E, immunohistochemistry (IHC), or *in situ* hybridization (ISH) staining.

### *In situ* hybridization

ISH on FFPE tissue was performed on the Ventana Discovery Ultra platform for IHC and ISH (Basel, Switzerland) based on the “DAKO” protocol by Jorgensen et al. (30). The same method has been used in previous publications by our group (31, 32). Buffers and detection reagents were purchased from Roche^®^ (Basel, Switzerland).

Protocol in short: Slides were baked at 60°C overnight and then transferred to the Discovery Ultra for ISH staining. Sections were deparaffinized at 68°C for three cycles in Ventana EZ buffer. Heat retrieval was performed at 95°C with Discovery Cell Conditioning Solution (CC1) for 40 minutes to make access for the probes. Optimized concentrations of probe controls and target miR probes were manually applied (miR-20a-5p, 50 nM; and miR-17-5p, 20 nM). The hybridization reaction was carried out for 60 minutes at 54°C for miR-17-5p and 40°C for miR-20a-5p, followed by two stringency washes with 2.0X SSC buffer. Possible unspecific bindings were blocked with AB blocking solution for 16 minutes. Alkaline phosphatase-conjugated anti-DIG (Anti-DIG-AP) was incubated for 20 minutes for immunologic detection. Substrate enzymatic reactions were carried out using NBT/BCIP for 60–120 minutes to give a blue precipitate. The slides were counterstained with Nuclear Fast Red for contrast staining. Slides were then dehydrated through an increasing gradient of ethanol solutions to xylene and then mounted using a Histokitt mounting medium.

The good sensitivity level of the ISH method and minimal RNA degradation in tissue was confirmed using U6, snRNA control probe at a concentration of 1.5 nM. A scramble miR negative control probe at 10 nM indicated no unspecific staining from reagents or tissues. MicroRNA expression in tissues other than Non-Small cell lung cancer (NSCLC) was also confirmed using a multi-tissue TMA control. Optimizations of temperatures, time, and concentrations were conducted for each probe and reagent.

### Scoring methods

The TMA slides were digitized using a Panoramic 250 Flash III slide scanner (3DHistech, Budapest, Hungary) and processed using QuPath version 0.1.2 (33). Briefly, 1) each slide was preprocessed according to Bankhead et al.; 2) tissue cores were assigned to the corresponding patient ID; 3) the tissue within each TMA core was identified and tiled into 20 × 20-µm tiles; 4) a classifier for each miR, separating tumor, and others (stroma, necrosis, etc.) was constructed; 5) the classifier was applied to all TMA cores; and 6) the median marker intensity in the tumor compartments for each patient was calculated and exported for further analyses.

### Cell line studies

The functional properties of miR-17-5p or miR-20a-5p were evaluated in two different human epithelial lung cancer cell lines: the squamous cell carcinoma H520 (ATCC^®^ HTB-182) (34) and the adenocarcinoma A549 (ATCC^®^ CCL-185) (35). Cell lines were authenticated at the Forensic Center at the UiT The Arctic University of Norway using Short tandem repeat (STR)-based DNA typing (PowerPlex 16HS kit from Promega, Promega, Madison, USA). For all experiments, H520 and A549 were below passages 60 and 20, respectively.

### Transfection

For all experiments, cells were transiently transfected with either 10 µM hsa-miR-17-5p Pre-miR™ miRNA Precursor (catalog# PM12412; Thermo Fisher Scientific, IL, USA) or hsa-miR-20a-5p Pre-miR™ miRNA Precursor (catalog# AM17100, Thermo Fisher Scientific, USA).

To assess the transfection efficacy, miRs were transfected alongside the Cy3™ Dye-Labeled Pre-miR Negative Control #1 (catalog# AM17120, Thermo Fisher Scientific, USA) using the transfection reagent Lipofectamine™ RNAiMAX (catalog# 13778075, Thermo Fisher Scientific, USA). Transfected Cy3™ Dye-Labeled Pre-miR Negative Control emits fluorescent light when exposed to UV light, and using a fluorescence microscope, the transfection efficiency of our transfections was evaluated to be between 80% and 95% (31).

### Proliferation assay

For the colorimetric proliferation assay, 5 × 10^3^ cells/well were cultured in 96-well plates. At different time points, cells were incubated with 12 mM of [3-(4,5-dimethylthiazol-2-yl)-2,5-diphenyltetrazolium bromide] (MTT; 5 mg/mL) (catalog# M6494, Invitrogen, OR, USA) for 4 h at 37°C. The formazan crystals produced were solubilized by the addition of 0.01 M HCl/sodium dodecyl sulfate (SDS) (catalog# 28312; Thermo Fisher Scientific, IL, USA) and mixed thoroughly using the pipette. The cells were incubated at 37°C overnight to dissolve the formazan, and the absorbance was measured in the CLARIOstar^®^ plate reader (BMG Labtech, Ortenberg, Germany) at 570 nm.

### Invasion assay

The detailed methodology of the invasion assay has been previously reported (36). In brief, cells were seeded in ThinceRt chambers (Greiner Bio-One, Kremsmünster, Austria) and transfected for 48 h at 37°C. The membranes containing invading cells (under the surface of the membrane) were photographed using an inverted optical microscope. Three random microscope fields were selected for analysis. The area of invading cells was determined using the ImageJ software, and results were plotted (mean ± SEM of three independent experiments) in relation to the control (C = 1).

### Wound healing assay

Detailed methodology has been previously reported (36). Cells were incubated in a culture medium containing mitomycin C (10 µg/L) to avoid cell proliferation, “wounded” using a 200-µL sterile pipette tip, and then washed to remove detached cells and debris. After 4 h, the cells were transfected for 24 h at 37°C. Photographs of the same area of the wound were taken at 0 and 24 h. Images were captured using an inverted optical microscope and analyzed using the Micrometrics SE Premium 4 software. The areas that were occupied by migrating cells after 24 h of incubation (control and transfected cells) were calculated by subtracting the background levels at 0 h. Results were plotted (mean ± SEM) in relation to control (C = 1). Images are representative of three different experiments.

### Statistics

SPSS 28.0 (Chicago, IL, USA) was used for all statistical analyses. The mean miR expression across TMA cores was normalized to values between 0 and 100 and divided into high and low expression using the mean score as a cut-off. Correlations were analyzed using Spearman’s rank coefficient. For survival analyses, the primary endpoint used was disease-specific survival (DSS), defined as the time from the date of surgery to lung cancer death. Univariate survival curves were estimated using the Kaplan–Meier method, and the statistical significance between survival curves was assessed using the log-rank test. Curves were terminated when less than 10% of the patients were at risk. Clinicopathological variables associated with significant prognostic values from the univariate analyses and the miRs were entered in the multivariate analyses. For multivariate analyses, the backward conditional Cox regression analysis was used. Probability for stepwise entry and step removal was set to 0.05 and 0.10, respectively. p-Values < 0.05 were considered statistically significant for all analyses.

## Results

### Patient characteristics

Clinicopathological and demographic variables and their impact on DSS are presented in **Table 1**. Age at diagnosis ranged from 28 to 85 years, with a median of 67. Of the patients, 68% were male, and 96% of the patients were current or previous smokers.

**Table 1:** Associations between clinicopathological variables and disease-specific survival (n = 553, univariate analyses; log-rank test) with unadjusted hazard ratios (Cox regression analyses).

Variable	N (%)	5-year (%)	Median (months)	Unadjusted HR (95% CI)	P
Age					0.656
<65	234 (42.3)	58	127	1	
>65	319 (57.7)	58	NR	0.94 (0.72–1.23)	
Sex					0.025
Female	180 (32.5)	64	190	1	
Male	373 (67.5)	55	91	1.39 (1.04–1.86)	
ECOG performance status					0.009
0	324 (58.6)	63	235	1	
1	191 (34.5)	52	70	1.51 (1.15.1.99)	
2	38 (6.9)	52	NR	1.46 (0.82–2.59)	
Weight loss					0.958
<10%	497 (89.9)	58	190	1	
>10%	55 (9.9)	59	NR	1.01 (0.64–1.60)	
Histology					0.241
LUSC	307 (55.5)	64	235	1	
LUAD	239 (43.2)	52	73	1.25 (0.96–1.63)	
Other	7 (1.3)	67	NR	0.95 (0.24–3.86)	
T-stage					<0.001
T1	180 (32.5)	72	235	1	
T2	208 (37.6)	54	83	1.87 (1.33–2.63)	
T3	104 (18.8)	56	NR	1.69 (1.12–2.54)	
T4	61 (11.0)	31	21	3.46 (2.26–5.30)	
N-stage					<0.001
N0	379 (68.5)	70	235	1	
N+	174 (31.5)	32	25	3.25 (2.49–4.23)	
P-stage					<0.001
I	232 (42.0)	74	235	1	
II	185 (33.4)	59	114	1.70 (1.22–2.38)	
IIIA + IIIB	136 (24.6)	28	21	4.14 (2.99–5.75)	
Surgical procedure					<0.001
Wedge/lobectomy	411 (74.3)	64	235	1	
Pulmonectomy	142 (25.7)	42	30	2.03 (1.54–2.68)	
Free margins					0.105
Free	506 (91.5)	59	190	1	
Not free	47 (8.5)	47	57	1.43 (0.93–2.20)	
Vascular infiltration					<0.001
No	453 (82.0)	62	235	1	
Yes	97 (17.5)	38	35	1.94 (1.42–2.66)	
Missing	3 (0.5)				
MiR-17-5p					0.275
Low expression	230 (41.6)	62	190	1	
High expression	230 (41.6)	54	114	1.17 (0.88–1.57)	
Missing	93 (16.8)				
MiR-20a-5p					0.137
Low expression	235 (42.5)	55	88	1	
High expression	235 (42.5)	61	235	0.80 (0.60–1.07)	
Missing	83 (15.0)				

ECOG, Eastern Cooperative Oncology Group Performance Status Scale; T-stage, tumor stage; N-stage, node stage; P-stage, pathological stage.

Bold values are significant at p ≤ (less than equal to) 0.05.

### Evaluation of miR-17-5p and miR-20a-5p expression and TMA staining

**Figure 1:**
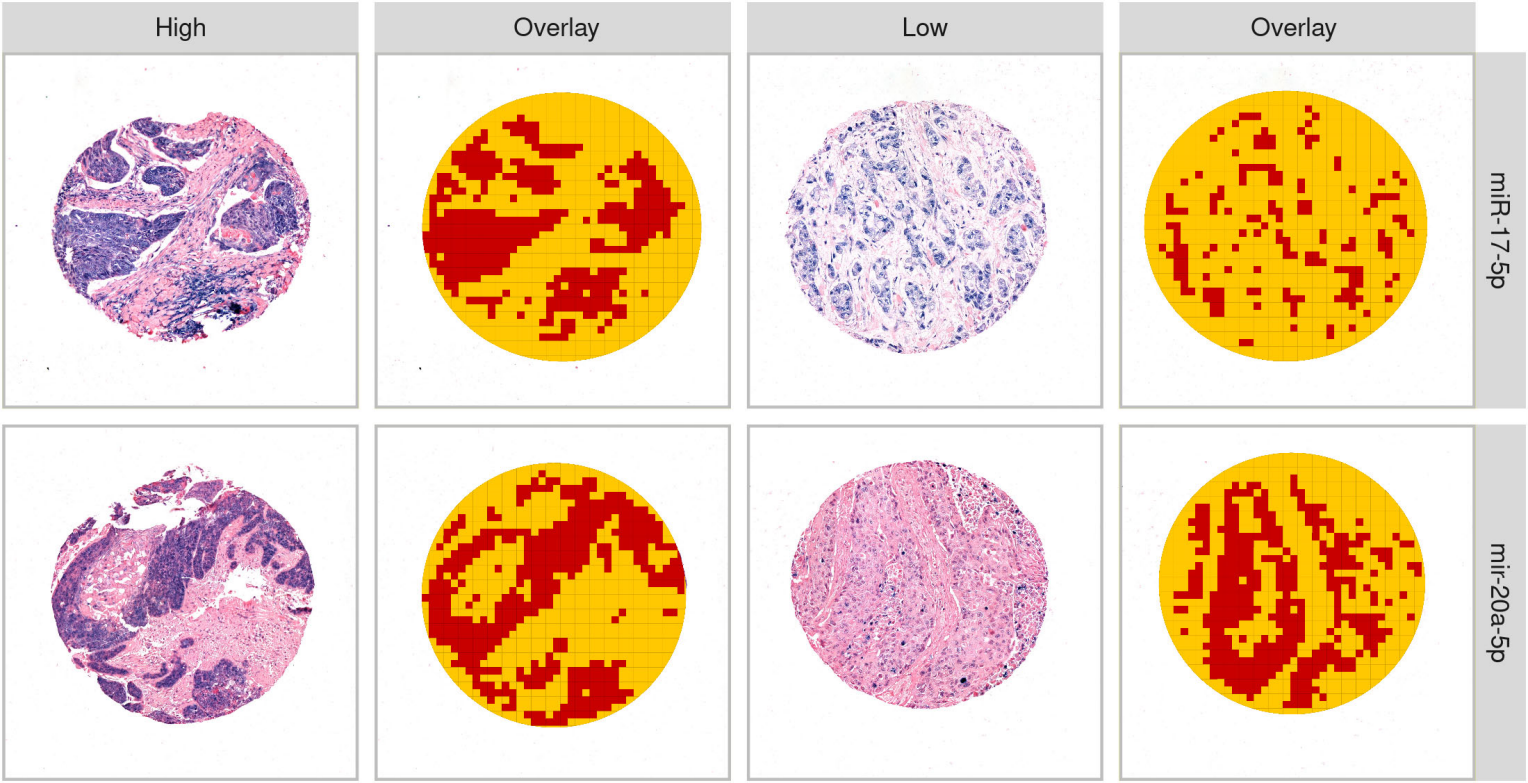
Examples of high and low expression of miR-17-5p and miR-20a-5p. Columns 2 and 4 give examples of the tissue classifier differentiating “tumor” (red) and “other” (yellow).

MiR-17-5p and miR-20a-5p were both predominantly expressed by tumor epithelial cells and in immune cell aggregates. When present, staining was found in both the nucleus and cytoplasm (**Figure 1**). Out of the 553 patients available for analysis, some were excluded based on damaged or missing TMA cores, poor staining or tissue quality, or no/few viable tumor cells identified (reviewed by pathologist LTB). After curation, a total of 460 and 470 patients were available for miR-17-5p and miR-20a-5p scoring, respectively. Representative QuPath exports of single cores at higher magnification are provided in the **Supplementary Material**.

### Scoring of ISH

The classifier for miR-17-5p was trained on a total of 13,332 tiles (4,428 and 8,904 defined as tumor and “other”, respectively), while the classifier for miR-20a-5p was trained on 46,181 tiles (19,382 and 26,799 defined as tumor and “other”, respectively). Both classifiers demonstrated an estimated precision of 98% for separating tumors from other tissues, using 20% of the available tiles as a test set. Manual evaluation revealed that, in some cases, the classifier was inaccurate, especially when differentiating between tumor cells and saturated miR-stained immune cells and when tumor cores were faintly stained. Examples of tumor tissue detected by the QuPath classifiers are shown in **Figure 1**. The mean and median of the normalized values of miR-17-5p and miR-20a-5p expression in the tumor compartment are found in **Supplementary Figure S1**.

### Correlations

The correlations between miR expression and clinicopathological variables were weak (r < 0.250) or non-significant. Correlations between the miRs and other tested biomarkers in our cohort were weak, with the exception of positive correlations with Her3 (miR-17-5p r = 0.348 and miR-20a-5p r = 0.286), MCT1 and MCT2 (miR-20a-5p r = 0.255 and 0.258), MMP9 (miR-17-5p r = 0.292), VEGFR1 (miR-17-5p r = 0.250), and CTLA4 (miR-17-5p r = 0.264).

Due to the strong prognostic impact of lymphocytes in our cohort, we specifically investigated the association between the miRs and immune cells with emphasis on lung squamous cell carcinoma (LUSC) patients. However, we did not observe any significant correlations between the investigated miRs and immune cell markers previously investigated in the cohort.

### Univariate analyses

**Figure 2:**
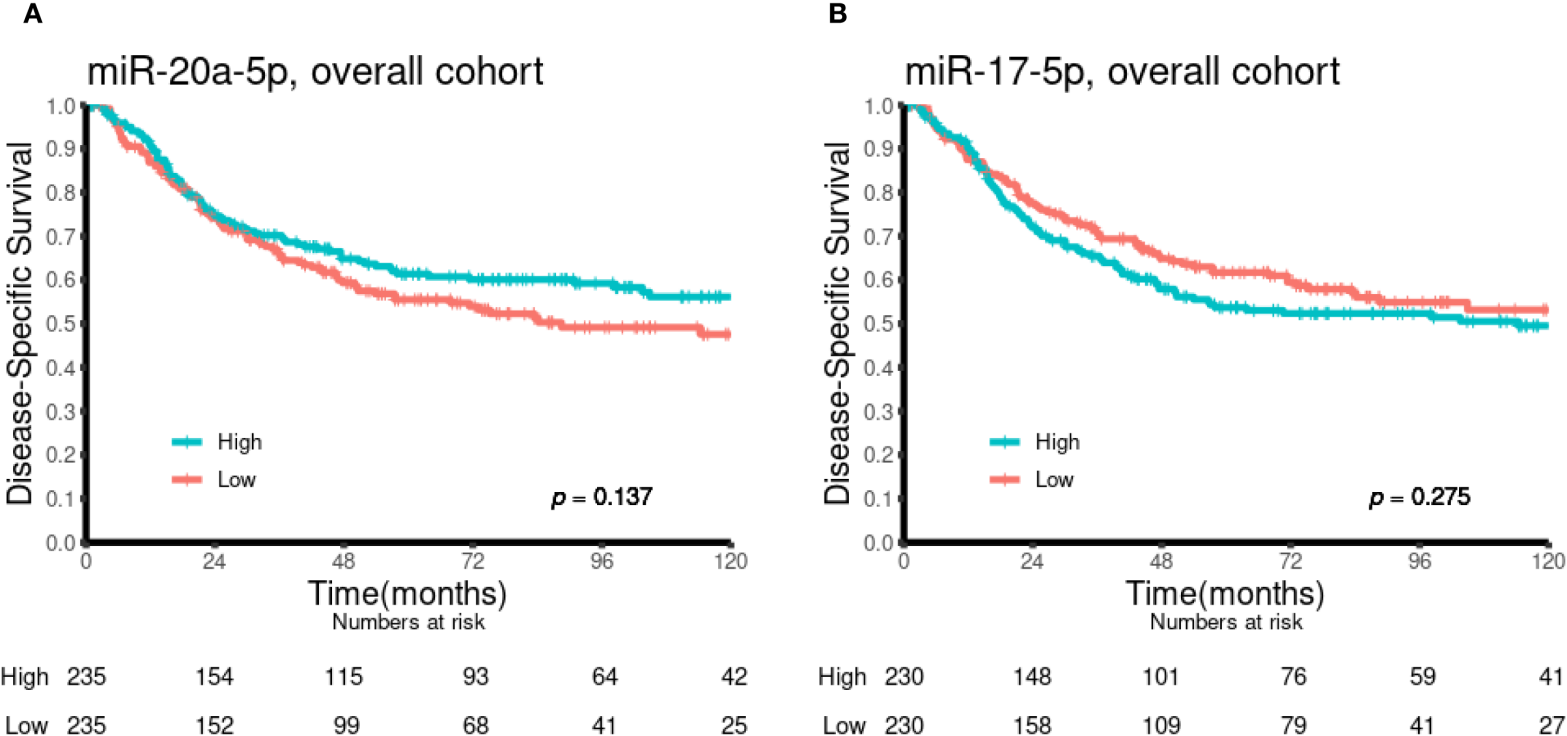
Kaplan–Meier curves illustrating the association between miR-20a-5p **(A)** and miR-17-5p **(B)** expression and disease-specific survival in the overall cohort (n = 553).

**Figure 3:**
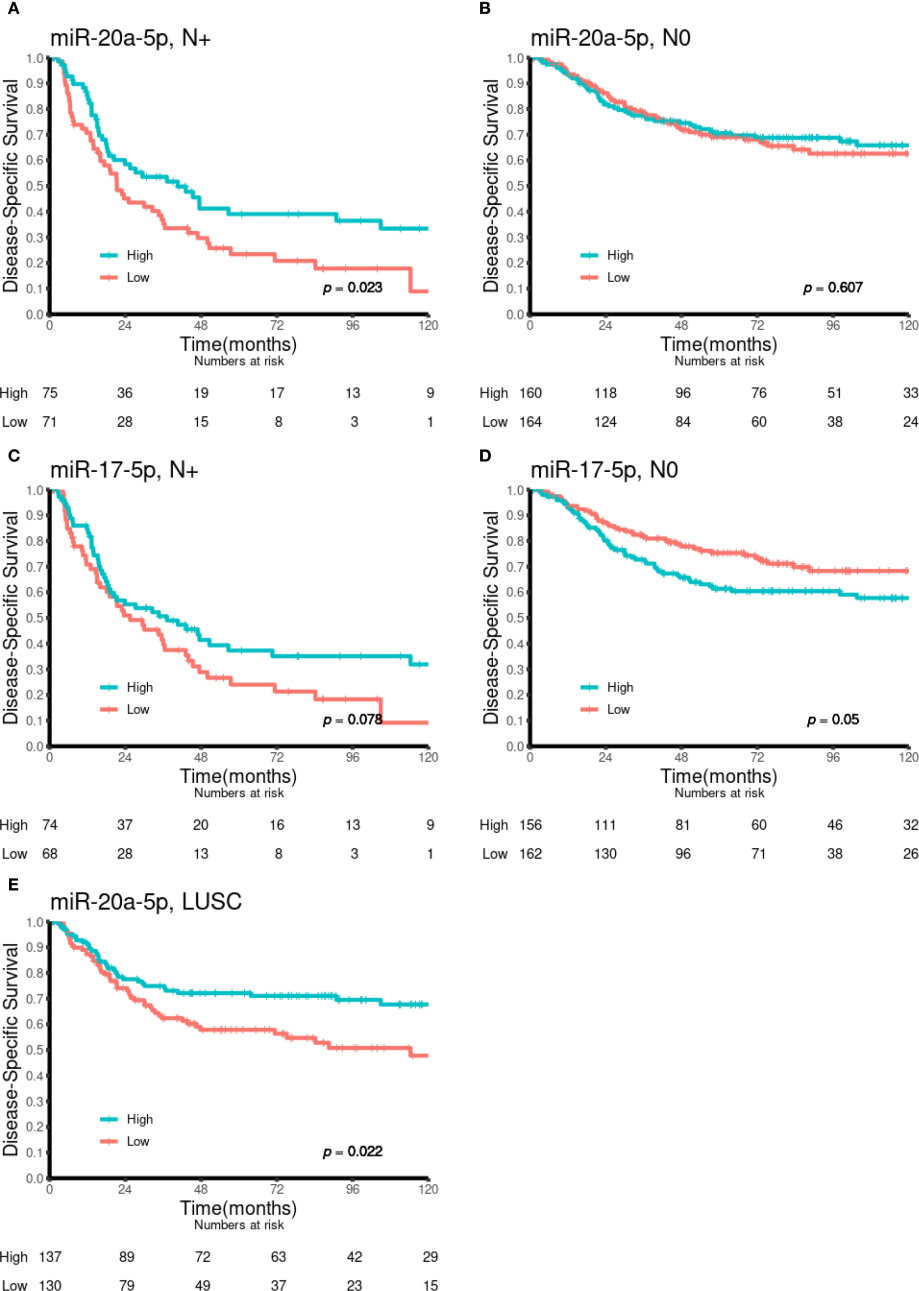
Kaplan–Meier curves illustrating the association between miR-20a-5p and miR-17-5p expression and DSS in various patient subgroups. **(A)** MiR-20a-5p in N+, **(B)** miR-20a-5p in N0, **(C)** miR-17-5p in N+, **(D)** miR-17-5p in N0, and **(E)** miR-20a-5p in LUSC. DSS, disease-specific survival; LUSC, lung squamous cell carcinoma.

Univariate analyses of the dichotomized miR-17-5p and miR-20a-5p expression in the overall cohort and in selected subgroups are presented in **Table 1** and **Figures 2** and **3**. No significant prognostic impact according to either miR expression or DSS was observed in this population. In subgroup analyses, high expression of miR-20a-5p was significantly associated with increased DSS in patients with lymph node metastases (N+, p = 0.023) and lung squamous cell carcinoma histology (p = 0.022). For miR-17-5p, a similar trend was observed in N+ patients (p = 0.078), while high expression was significantly associated with reduced DSS in patients without lymph node metastases (N0, p = 0.050).

### Multivariate analyses

Multivariate analyses for miR-17-5p and miR-20a-5p are presented in **Tables 2** and **3**, respectively. In the multivariate analysis of the overall cohort, high expression of miR-20a-5p was an independent predictor of increased DSS (HR 0.73, 95% CI 0.54–0.99, p = 0.040). In subgroup analyses, high expression of miR-20a-5p was an independent predictor of increased DSS in N+ (HR 0.58, 95% CI 0.38–0.89, p = 0.014) and LUSC (HR 0.49, 95% CI 0.32–0.76, p = 0.002) patients.

**Table 2:** Multivariable models of miR-20a-5p as a prognostic marker of DSS in the overall cohort and in the LUSC and N+ subgroups (Cox proportional hazards test, n = 553, 307, and 174, respectively).

Variable	Model 1 (miR-20a-5p overall)			Model 2 (LUSC)			Model 3 (N+)		
Factor	Hazard ratio	95% CI	P	Hazard ratio	95% CI	P	HR	95% CI	P
Histology			**0.045**						
LUSC	1 (ref)								
LUAD	1.450	1.082–1.944	**0.013**						
Other	1.054	0.257–4.322	0.942						
ECOG			0.037						
0	1 (ref)								
1	1.468	1.081–1.992	**0.014**						
2	1.492	0.826–2.695	0.185						
N-stage			**<0.001**			**<0.001**			
N0	1 (ref)			1 (ref)					
N+	3.020	2.239–4.073		3.447	2.234–5.317				
T-stage			**<0.001**			**<0.001**			**0.035**
T1	1 (ref)			1 (ref)			1 (ref)		
T2	1.512	1.038–2.204	**0.031**	1.365	0.778–2.395	0.279	1.812	0.955–3.437	0.069
T3	1.229	0.764–1.978	0.396	1.071	0.546–2.102	0.842	2.422	1.168–5.023	**0.017**
T4	2.784	1.735–4.467	**<0.001**	3.389	1.757–6.534	**<0.001**	2.949	1.350–6.440	**0.007**
Vascular infiltration	1.584	1.114–2.252	**0.010**				1.564	0.984–2.485	0.059
MiR-20a-5p			**0.040**			**0.002**			**0.014**
Low	1 (ref)			1 (ref)			1 (ref)		
High	0.732	0.544–0.986		0.493	0.318–0.766		0.583	0.379–0.897	

ECOG, Eastern Cooperative Oncology Group Performance Status Scale; LUSC, lung squamous cell carcinoma; LUAD, lung adenocarcinoma; T-stage, tumor stage; N-stage, node stage; P-stage, pathological stage; DSS, disease-specific survival.

Bold values are significant at p ≤ (less than equal to) 0.05.

**Table 3:** Multivariable models of miR-17-5p as a prognostic marker of DSS in the overall cohort and in the N+ and N0 subgroups (Cox proportional hazards test, n = 553, 174, and 379, respectively).

Variable	Model 1 (miR-17 overall)			Model 2 (N+)			Model 3 (N0)		
Factor	Hazard ratio	95% CI	P	Hazard ratio	95% CI	P	HR	95% CI	P
Histology			**0.034**						**0.043**
LUSC	1 (ref)						1 (ref)		
LUAD	1.469	1.098–1.966	**0.010**				1.659	1.106–2.487	**0.014**
Other	1.036	0.253–4.242	0.961				0.755	0.101–5.640	0.784
ECOG			**0.048**						**0.012**
0	1 (ref)								
1	1.457	1.074–1.976	**0.015**				1.829	1.203–2.780	**0.005**
2	1.379	0.749–2.538	0.303				1.978	0.869–4.501	0.104
N-stage			**<0.001**						
N0	1 (ref)								
N+	2.801	2.081–3.771							
T-stage			**<0.001**			0.085			**<0.001**
T1	1 (ref)			1 (ref)			1 (ref)		
T2	1.387	0.958–2.008	0.083	1.469	0.796–2.711	0.219	1.285	0.796–2.076	0.305
T3	1.159	0.729–1.844	0.532	1.990	0.992–3.990	0.053	0.704	0.353–1.405	0.320
T4	2.588	1.637–4.090	**<0.001**	2.439	1.154–5.156	**0.020**	2.967	1.671–5.268	**<0.001**
Vascular infiltration	1.690	1.190–2.400	**0.003**	1.579	0.988–2.522	0.056	1.693	0.980–2.935	0.059
MiR-17-5p			0.685/NS			0.051			**0.035**
Low	1 (ref)			1 (ref)			1 (ref)		
High	1.063	0.791–1.428		0.653	0.426–1.002		1.545	1.032–2.312	

ECOG, Eastern Cooperative Oncology Group Performance Status Scale; LUSC, lung squamous cell carcinoma; LUAD, lung adenocarcinoma; T-stage, tumor stage; N-stage, node stage; P-stage, pathological stage; DSS, disease-specific survival.

Bold values are significant at p ≤ (less than equal to) 0.05.

MiR-17-5p was not associated with DSS in the overall cohort (**Table 3**, Model 1). In the subgroup analysis of N0 patients, high expression of miR-17-5p was an independent predictor of reduced DSS (HR 1.55, 95% CI 1.03–2.31, p = 0.035).

### Cell line studies

**Figure 4:**
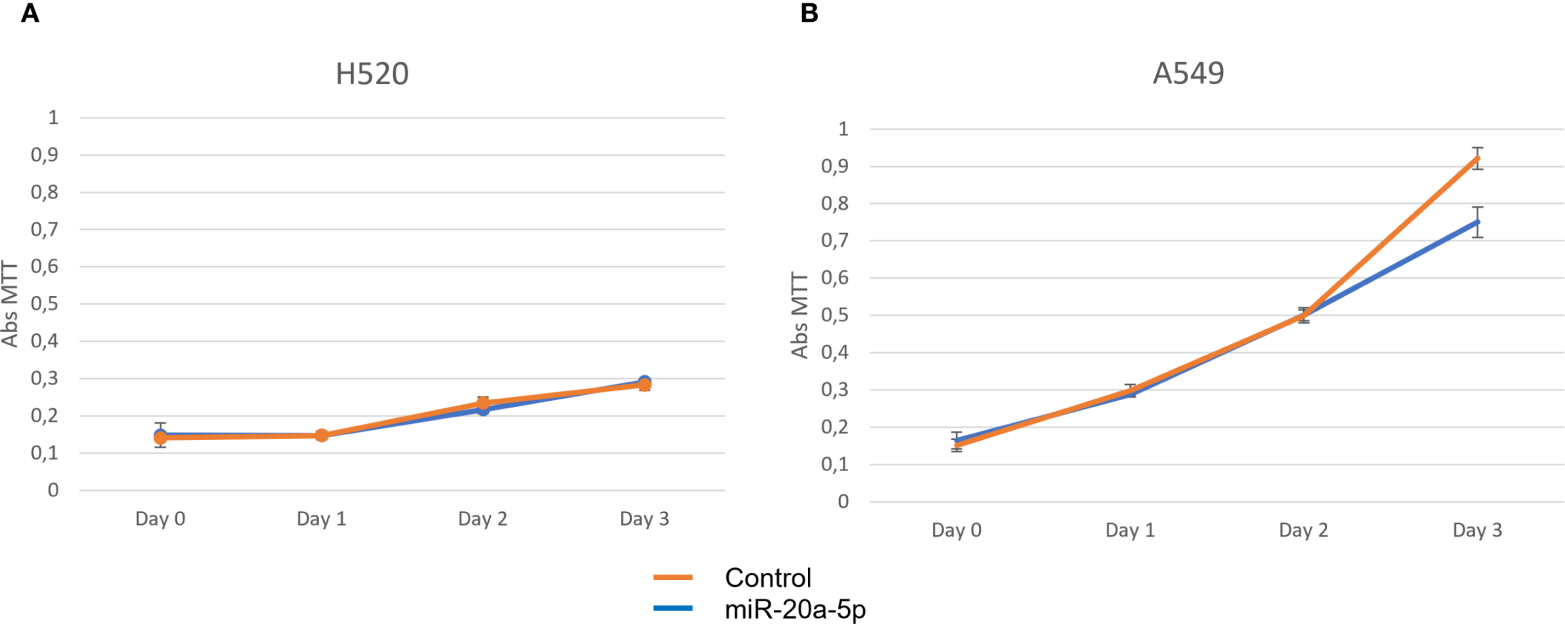
Proliferation assays for miR-20a-5p (blue) were measured in squamous cell carcinoma H520 **(A)** and adenocarcinoma A549 **(B)**, comparing cells transfected with miR-20a-5p (blue) to controls (orange). Images are representative of three different experiments.

**Figure 5:**
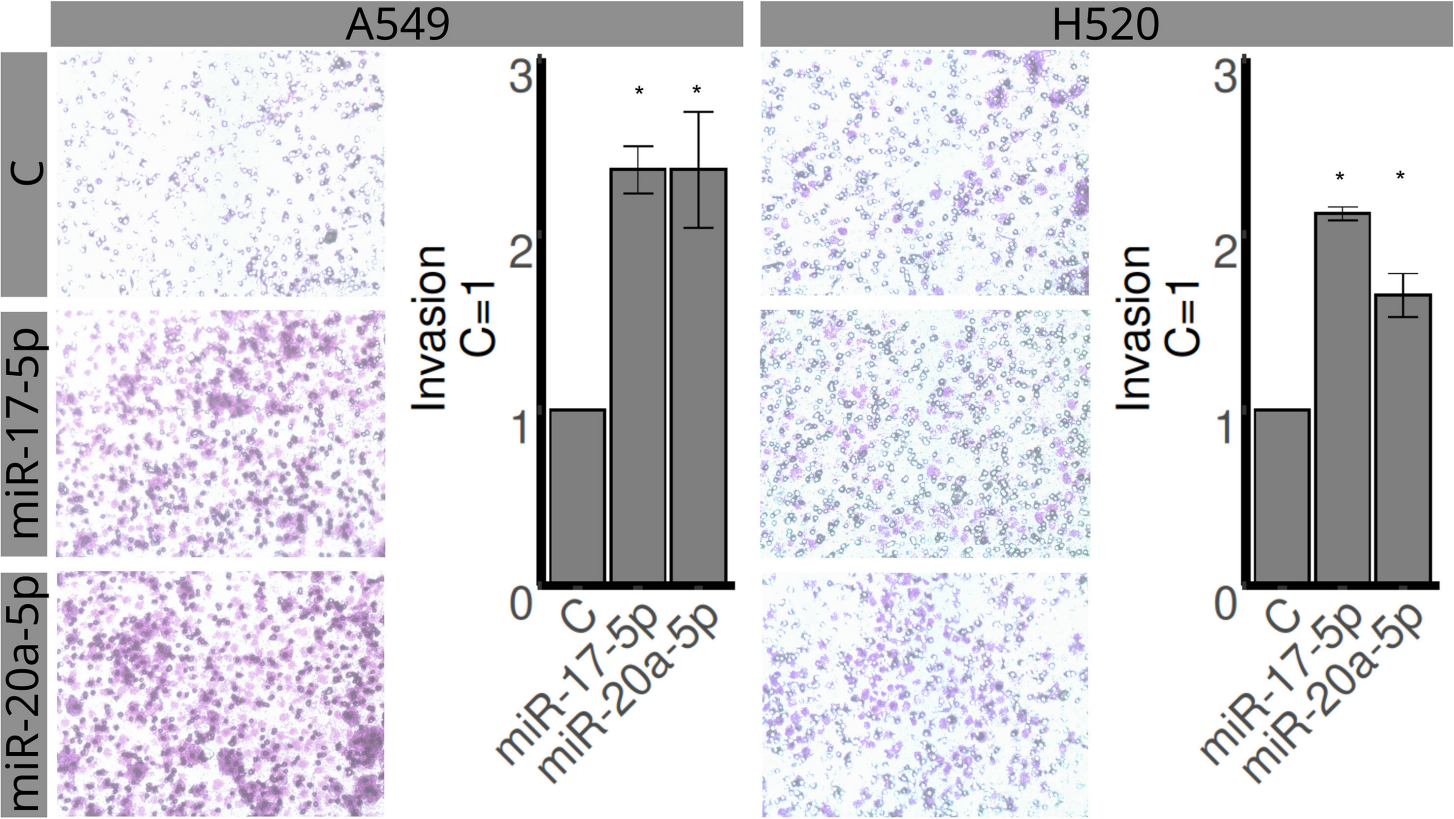
Effects of miR-17-5p and miR-20a-5p transfection on the invasion in A549 (left) and H520 (right) cell lines for 48 h. The upper row represents control, the middle row miR-17-5p, and the lower row miR-20a-5p. Images are representative of three different experiments. Bar graphs show quantification of invaded areas (mean ± SEM) in relation to control (C = 1). *Significantly different from control, C (p < 0.05, Student’s t-test).

**Figure 6:**
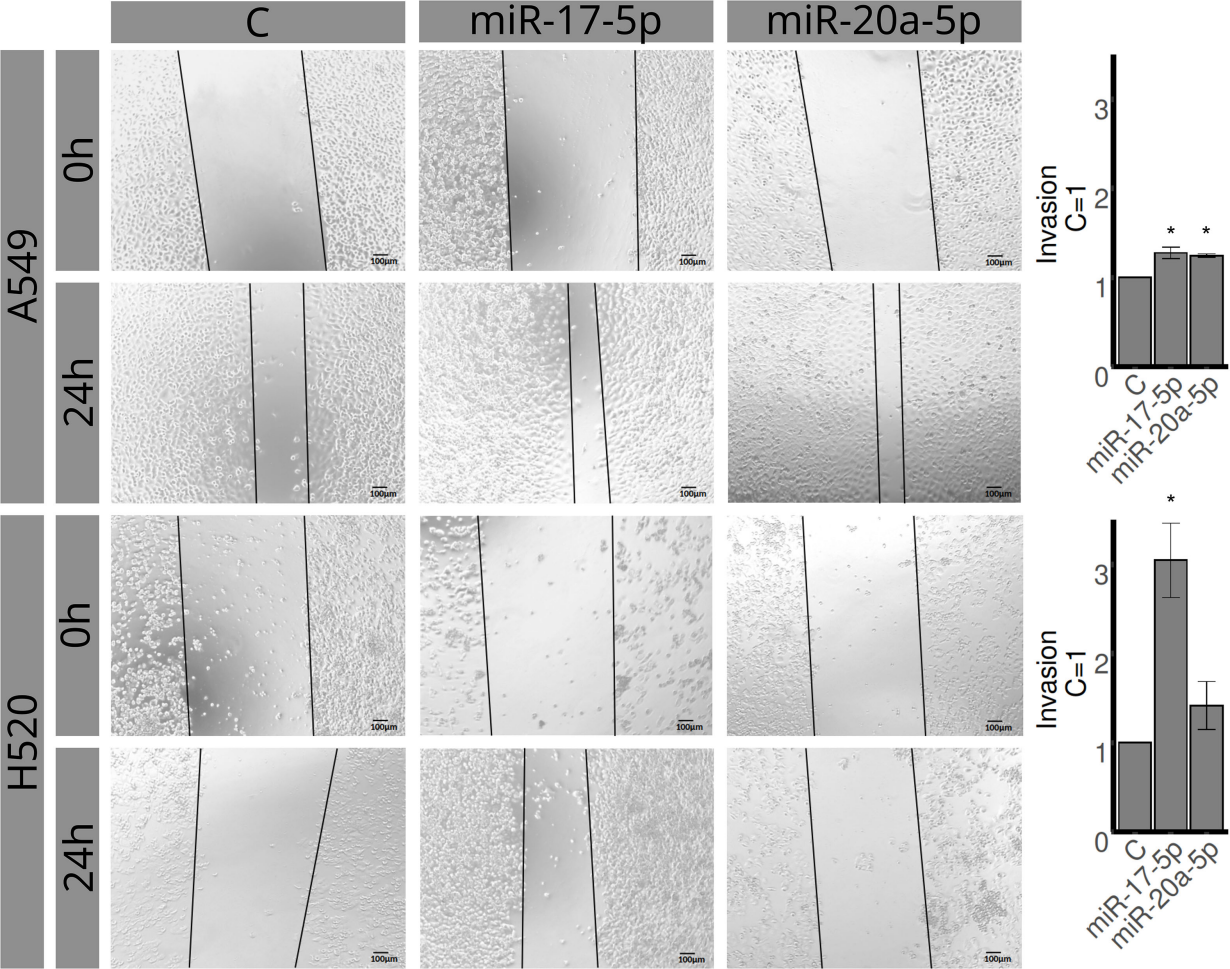
Effects of miR-17-5p and miR-20a-5p on migration in A549 (upper row, 0 and 24 h) and H520 (lower row, 0 and 24 h) cell lines. Columns represent control (left), miR-17-5p (middle), and miR-20a-5p (right). Images are representative of three different experiments. Bar graphs represent areas occupied by migrating cells after 24 h, calculated by subtracting baseline levels at 0 h, and plotted (mean ± SEM) in relation to control (C = 1). *Significantly different from control, C (p < 0.05, Student’s t-test).

The H520 and A549 cell lines were assessed for proliferation, invasion, and migration using MTT, transwell, and wound healing assays, respectively (**Figures 4**–**6**). Overexpression of miR-20a-5p led to reduced proliferation in the A549, but not in the H520 cell line (**Figure 4**), increased invasion in both cell lines (**Figure 5**), and increased migration in the A549 cell line (**Figure 6**). Overexpression of miR-17-5p did not lead to changes in proliferation in either cell line but induced increased invasion (**Figure 5**) and migration (**Figure 6**) in both cell lines.

## Discussion

We analyzed the expression of miR-17-5p and miR-20a-5p in a cohort of 553 consecutively resected NSCLC patients. To our knowledge, this is the first study to investigate miR-17-5p and miR-20a-5p in NSCLC using digital image analysis of *in situ* expression in the tumor compartment. In the overall cohort, we found no association between tumor expression of miR-17-5p and DSS, whereas high expression of miR-20a-5p was associated with a favorable prognosis (HR 0.732, 95% CI 0.544–0.986, p = 0.040). Moreover, subgroup analyses revealed that high expression of miR-20a-5p was associated with a positive prognosis in patients with LUSC (HR 0.49, 95% CI 0.32–0.76, p = 0.002) and in patients with lymph node metastases (N+, HR 0.58, 95% CI 0.38–0.89, p = 0.014). Interestingly, high expression of miR-17-5p was associated with poor DSS in patients without lymph node metastases (N0, HR 1.55, 95% CI 1.03–2.31, p = 0.035). Further, *in vitro* studies in the A549 lung adenocarcinoma (LUAD) and H520 LUSC cell lines revealed that overexpression of miR-17-5p did not change proliferation in either cell line but led to a significant increase in invasion and migration in both. Overexpression of miR-20a-5p resulted in reduced proliferation for A549 cells and an increase in invasion and migration in both cell lines.

In our study, high expression of miR-17-5p in N0 NSCLC patients was associated with shorter survival, while overexpression *in vitro* led to increased invasion and migration in two NSCLC cell lines. Together, our results indicate that high expression of miR-17-5p may drive NSCLC cells toward an invasive phenotype. Further corroborating miR-17-5p’s role in invasion, we observed no prognostic impact in N+ patients (an opposite trend was observed, p = 0.078), suggesting that its negative prognostic impact is abrogated once invasion is established. Our results align with several meta-analyses reporting that miR-17-5p (37–39) or the miR-17–92 cluster (40, 41) is associated with poor prognosis across several types of cancer. In NSCLC, Saito et al. reported on the prognostic impact of several miRs, including miR-17, using RT-PCR on snap-frozen tumor samples (42). They found that miR-17 was associated with poor survival in their discovery cohort of 89 patients but were unable to confirm this result in two independent cohorts. Contrary to our study, they did not distinguish between miR-17-3p and miR-17-5p, nor did they investigate the prognostic impact of miR-17-5p in N0 *vs*. N+ patients. Additionally, their independent cohorts comprised only 37 and 191 patients, and the largest of these consisted of 50% never-smokers whose lung cancers are known to be molecularly different from those of smokers. Moreover, several studies have investigated circulating miR-17-5p in NSCLC, found an association with advanced stage and shorter survival, and even proposed it as a diagnostic test for early detection/screening in Asian populations (14, 43–46). Results from studies in cell lines and mice are less clear. While some, like our study using A549 and H520 cells, report that overexpression of miR-17-5p leads to an invasive phenotype, other studies suggest that high levels of miR-17-5p lead to tumor suppression through inhibited proliferation and migration/invasion. For example, Chen et al. found that miR-17-5p was downregulated in most NSCLC cell lines and demonstrated that overexpression inhibited colony formation and migration/invasion in A549 cells and tumor growth in nude mice (16). Zheng et al. showed that silencing of the host gene MIR17HG led to reduced tumorigenicity and immune escape through the miR-17-5p/RUNX3 axis (23). Similarly, Huo et al. demonstrated that miR-17-5p acts as a tumor suppressor by targeting the lncRNA FGD5-AS1, reducing proliferation and migration in NSCLC cells (24). Others have also found miR-17-5p downregulated in A549 LUAD (47) and H226 LUSC cell lines (48). Moreover, context-dependent roles of miR-17-5p have been observed in several cancers (32, 49–51). Specifically, the immunomodulatory role of miR-17-5p, such as stimulating T-cell activity in melanoma, highlights its complexity in the tumor microenvironment (49).

Contrasting our findings for miR-17-5p, high expression of miR-20a-5p was associated with longer survival for NSCLC patients, with the strongest impact observed in patients with LUSC histology or N+ disease. Interestingly, we could not identify any previous studies reporting on the prognostic impact of miR-20a-5p expression in tumor tissue from NSCLC patients. Moreover, our cell line studies on miR-20a-5p yielded conflicting results. We observed that the transfection of miR-20a-5p reduced proliferation in A549 cells, while it induced invasion in both A549 and H520 cells and migration in A549 cells. These results indicate that miR-20a-5p exhibits both tumor-suppressive and tumor-promoting properties. Like in our *in vitro* studies, several previous experiments in cell lines and mice have reported conflicting findings. For example, Han et al. reported that miR-20a-5p inhibited angiogenesis in NSCLC (26), while Gong et al. demonstrated that miR-20a-5p can induce oncogenesis by targeting PTEN and promoting PD-L1 expression (25), indicating a role in immune evasion. The tumor-suppressive (32, 52–54) and oncogenic properties of miR-20a-5p (31, 55–57) have been reported for various cancers, reinforcing that its activity is probably tissue- and context-dependent.

Despite our promising results, the variable and sometimes opposing functional roles reported for miR-17-5p and miR-20a-5p highlight the need for further mechanistic studies before considering their use as clinical biomarkers or therapeutic targets in NSCLC. Importantly, their roles should be interpreted within the broader context of the miR-17–92 cluster and its paralogs (miR-106–25 and miR-106a-362), which encode 15 related miRs with overlapping but distinct targets (18). To realize their clinical potential, future research should aim to disentangle these complex interactions, including effects on the tumor microenvironment and on the immune system.

Although our digital pathology pipeline for miR expression quantification offers greater reproducibility and reduced subjectivity compared to semi-quantitative scoring, certain limitations remain. The tissue classifier occasionally failed to distinguish faintly stained tumor cells from stroma and to accurately classify densely stained immune cell clusters. These challenges underscore the need for further optimization or the integration of a human-in-the-loop approach before clinical implementation. Moreover, as this was a hypothesis-generating study, we did not adjust for multiple testing, acknowledging an increased risk of type I errors. Therefore, independent validation in large retrospective or prospective cohorts is essential before translating these findings into clinical practice.

## Conclusions

In our large NSCLC cohort, high miR-17-5p expression predicted shorter survival for N0 patients, whereas high miR-20a-5p expression predicted longer survival, especially in the LUSC and N+ subgroups. *In vitro*, both miRs promoted invasion, while results on migration and proliferation were less clear. Based on our results, we hypothesize that miR-17-5p is linked to early invasive transformation, while miR-20a-5p induces tumor suppression under certain conditions. These findings highlight the biological complexity of the miR-17–92 cluster, and the need for careful interpretation and further studies before therapeutic targeting is considered. A deeper understanding of these miRs could enable their use in prognostication and treatment stratification in NSCLC.

## Data Availability

The datasets presented in this article are not readily available because Data will be shared upon reasonable request to the corresponding author. Requests to access the datasets should be directed to dny006@uit.no.
