# Supplementary material for: Prognostic impact of miR-17-5p and miR-20a-5p in NSCLC diverge in subgroups according to lymph node status
Source: Front Oncol. 2025 Sep 25;15:1606933. doi: 10.3389/fonc.2025.1606933 (PMC12507588; doi:10.3389/fonc.2025.1606933)
Supplement: Supplementary file 1 [file DataSheet1.docx]

**Supplementary**

**Fig. S1.** Mean and median values of normalized score of miR-17-5p and miR-20a-5p


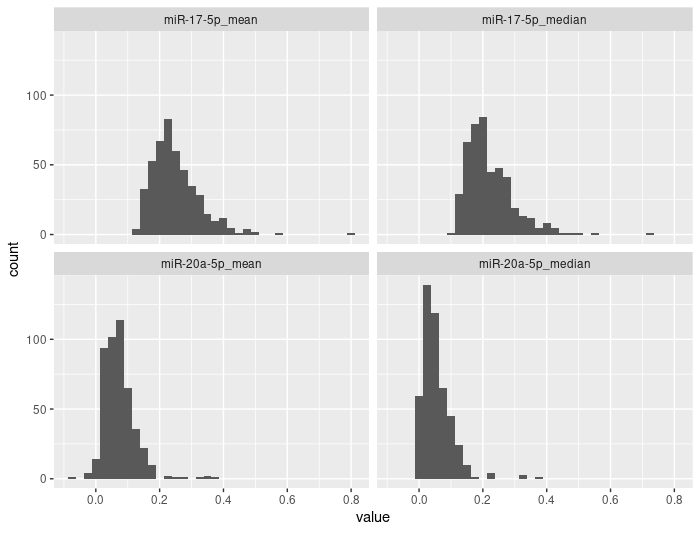


**S2. Qupath exports of representative single cores**

**
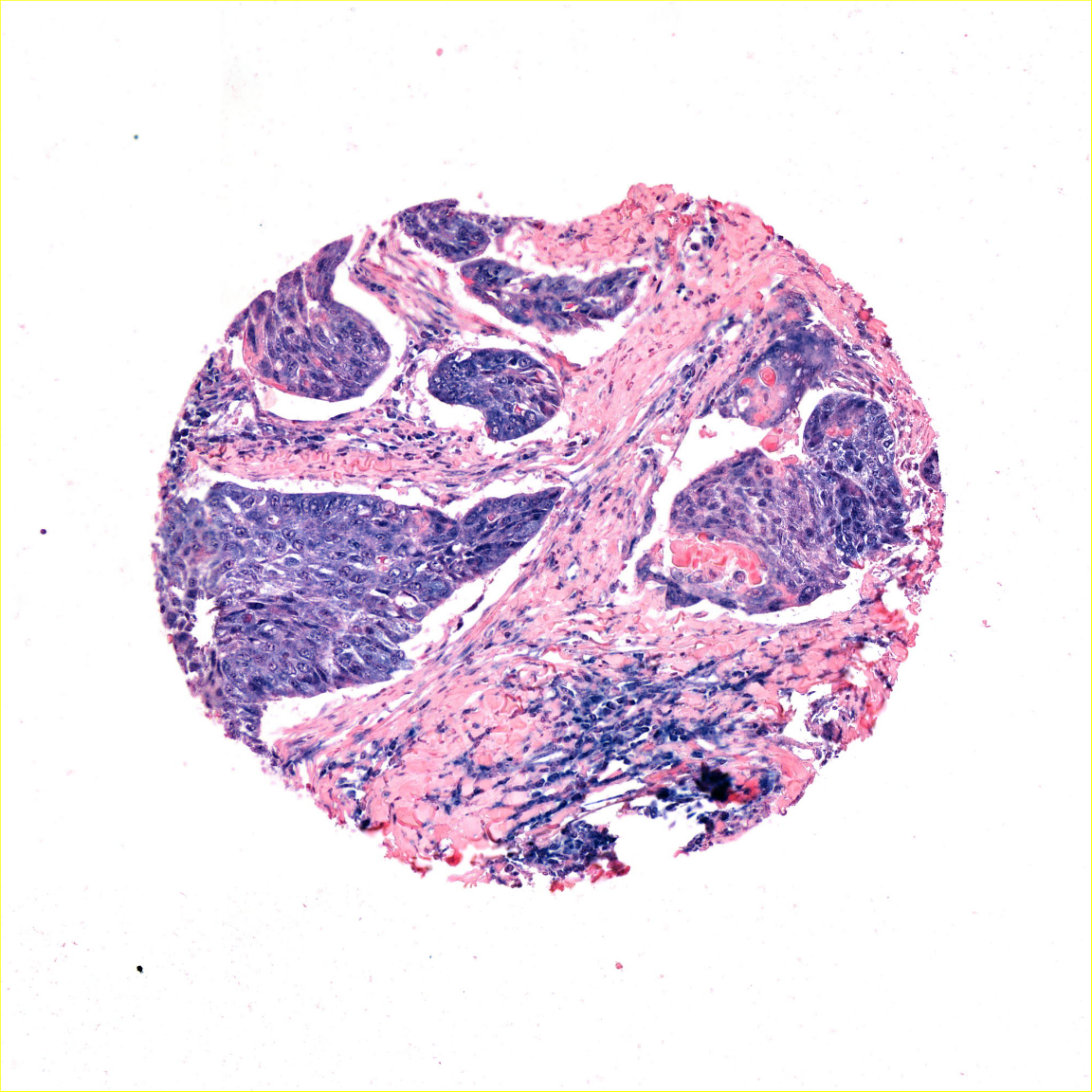

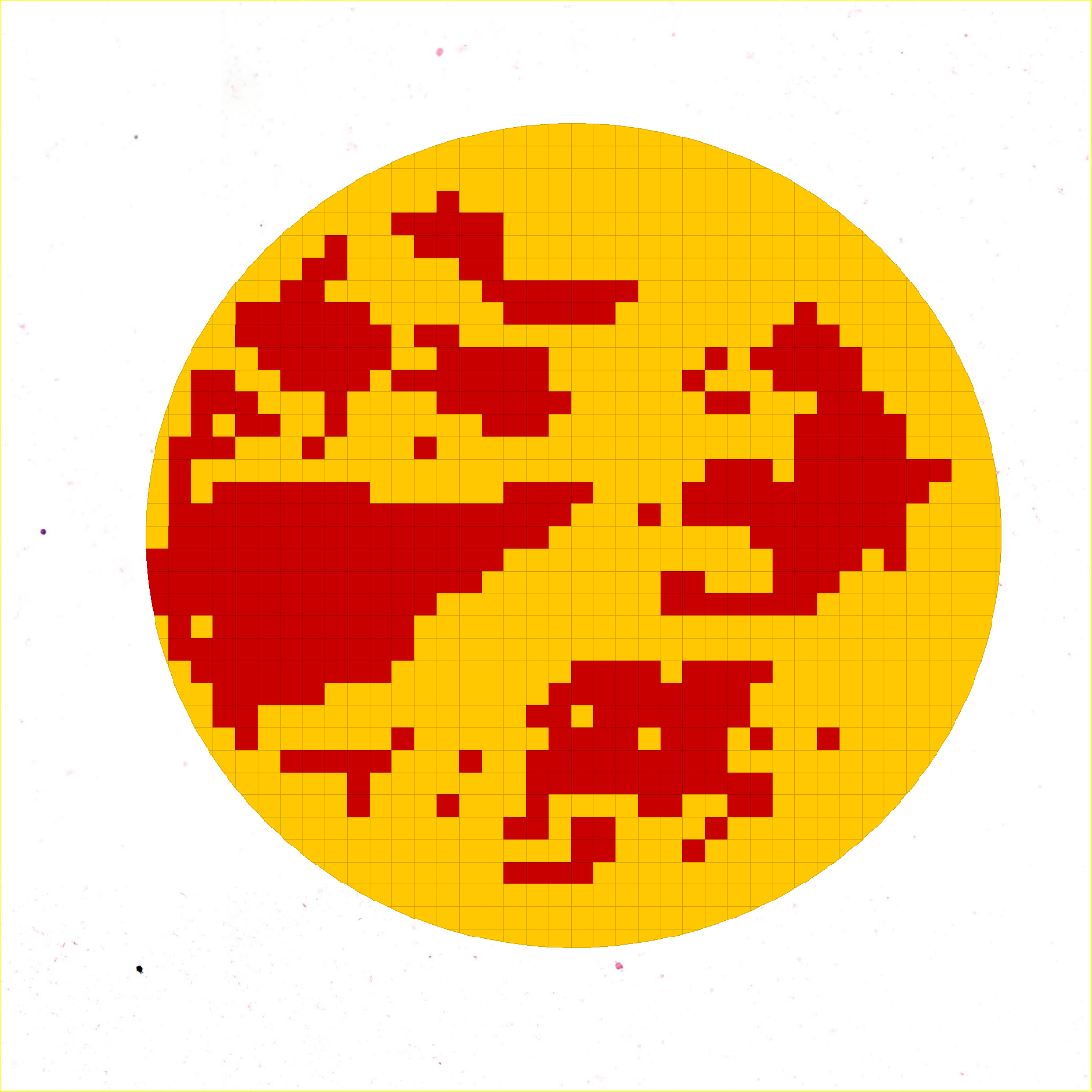

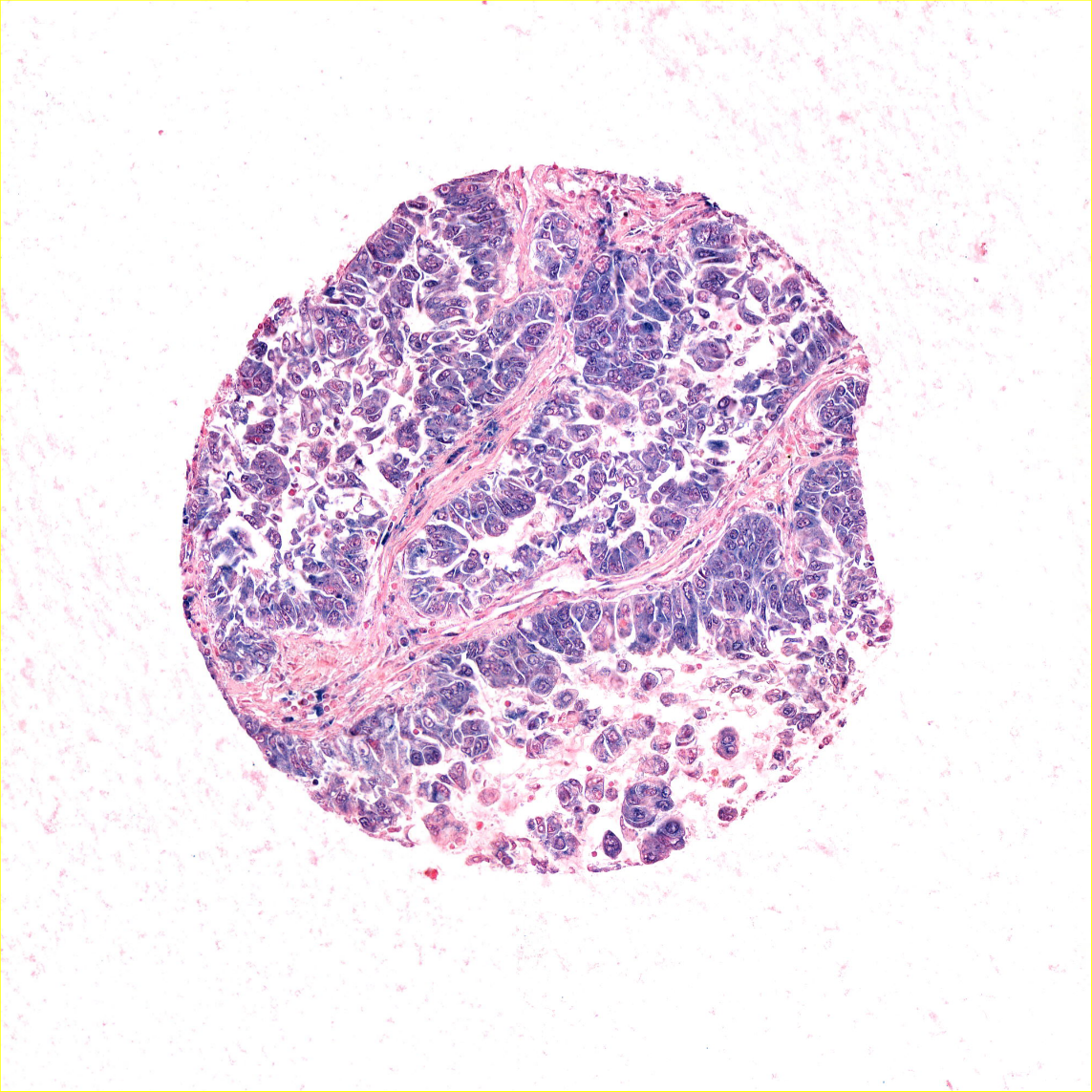

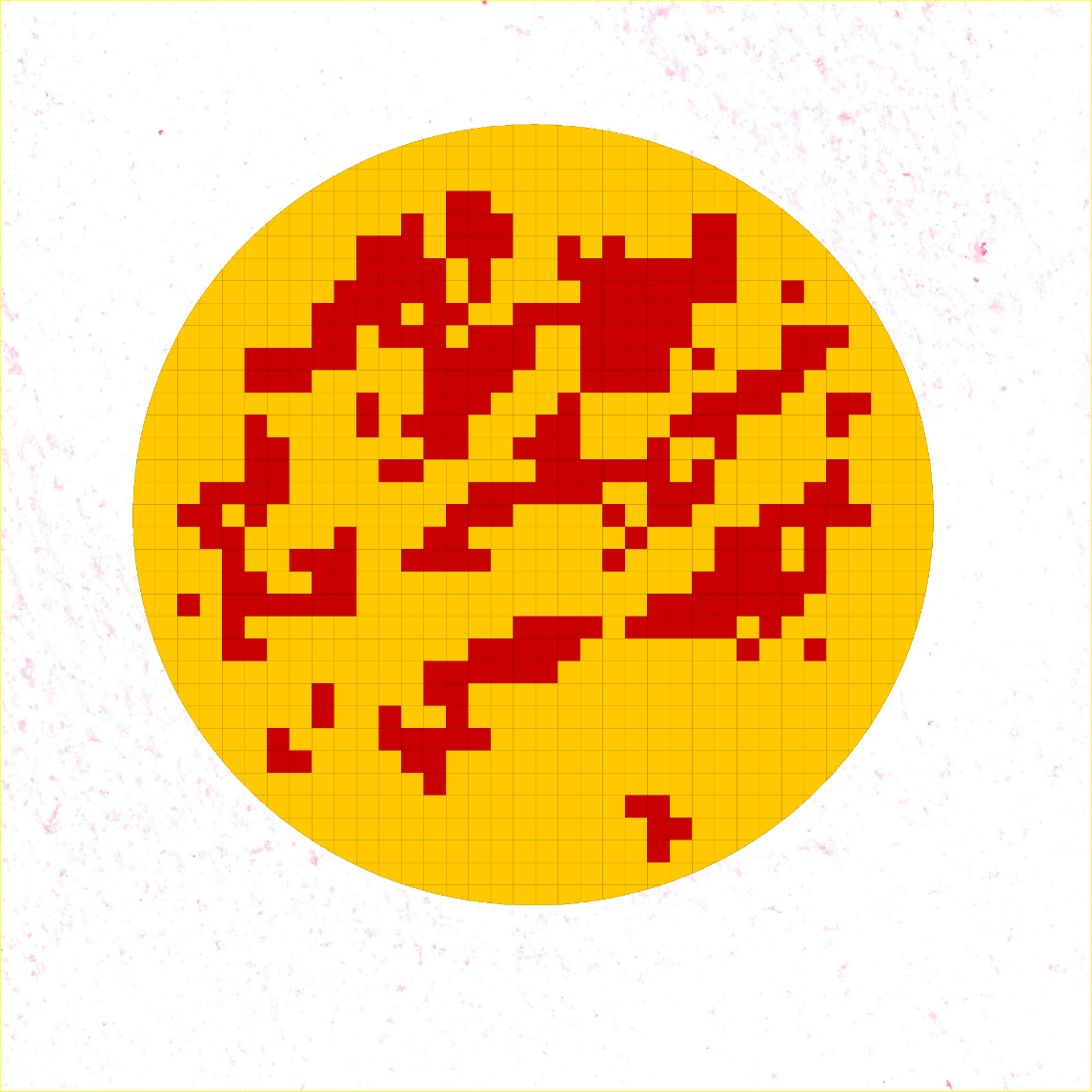

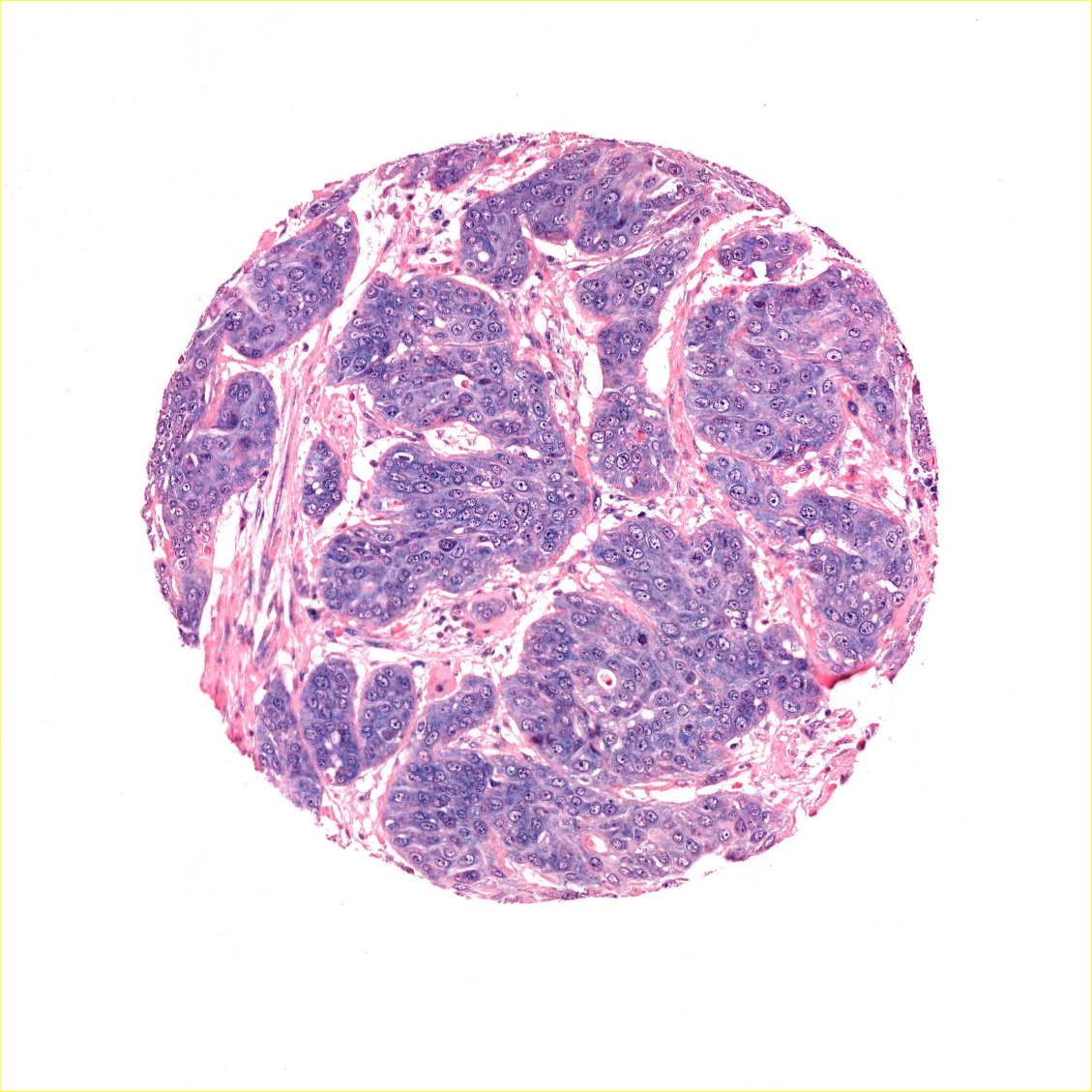

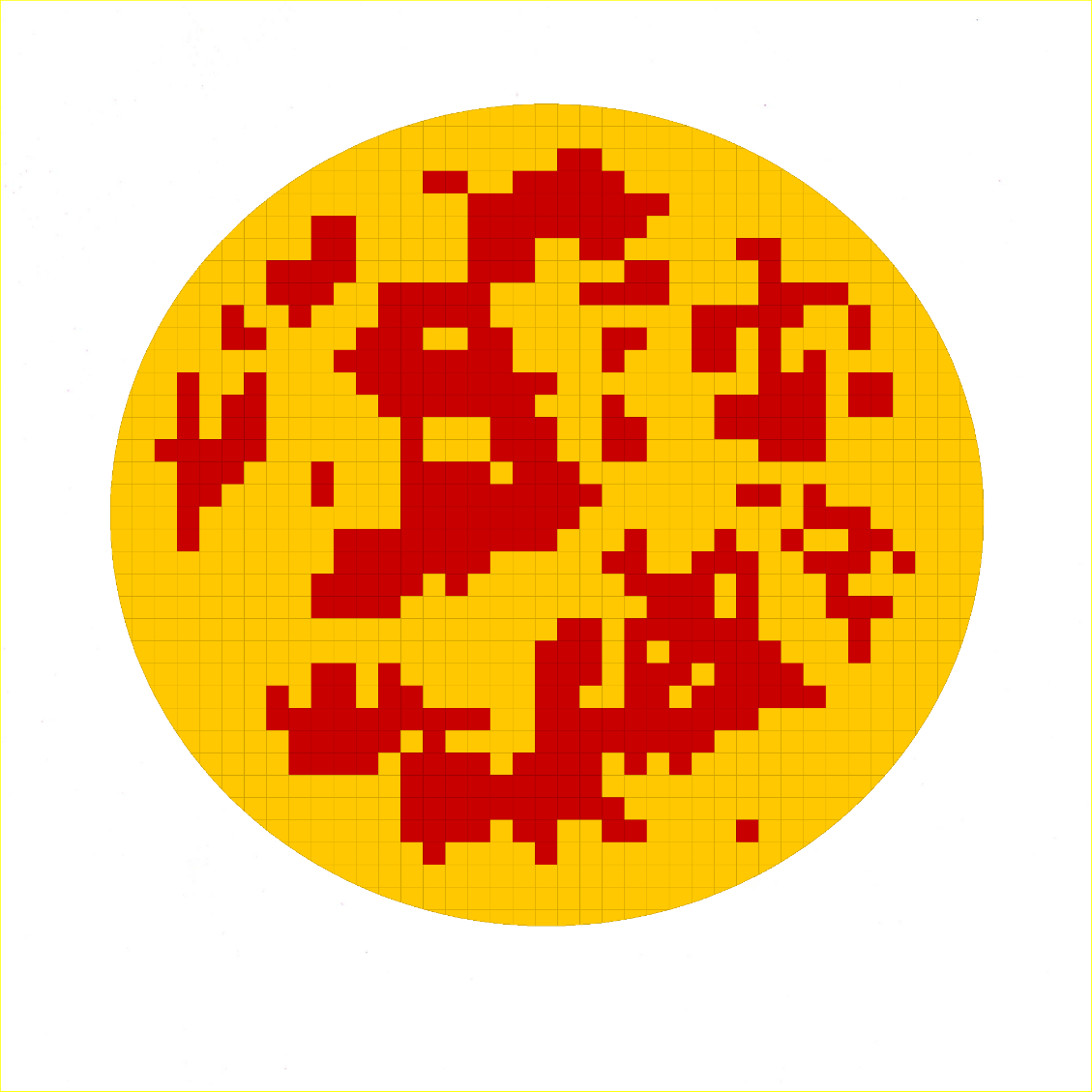
**
